# Biomechanical Assessment Using MRI of Custom‐Made Bovine Pericardial Tubes as Potential Aortic Replacements

**DOI:** 10.1155/rrp/6214002

**Published:** 2026-09-01

**Authors:** Abdulrahman Alblowi, Pierre-André Vuissoz, Siyu Lin, Olivier Bouchot, Thomas Decourselle, Jacques Felblinger, Nicla Settembre, Alain Lalande, Serguei Malikov

**Affiliations:** ^1^ Department of Vascular and Endovascular Surgery, Nancy Regional University Hospital, University of Lorraine, INSERM UMRS 1116 DCAC, Nancy, France, univ-lorraine.fr; ^2^ IADI U1254, INSERM and Université de Lorraine, Nancy, France; ^3^ ICMUB CNRS UMR 6302, Université Bourgogne Europe, Dijon 21000, France; ^4^ Division of Vascular and Endovascular Surgery, Department of General Surgery, King Fahad Hospital of the University, College of Medicine, Imam Abdulrahman Bin Faisal University, Dammam, Saudi Arabia, iau.edu.sa; ^5^ University Hospital of Dijon, Dijon, France, chu-dijon.fr; ^6^ CASIS—Cardiac Simulation & Imaging Software SAS, Quetigny, France

**Keywords:** custom-made bovine pericardial tube, distensibility, MRI, strain percentage, velocity, xenograft material

## Abstract

**Introduction:**

Autologous materials for vessel replacement in infected fields have high morbidity and mortality rates. Custom‐made bovine pericardial tubes (CMBPTs) offer a promising alternative with advantages such as reliability, strength, and availability. However, limited biomechanical data hinder predictions of in vivo performance. This study evaluates the physiological strain and distensibility of CMBPTs in vitro under simulated abdominal aortic flow using magnetic resonance imaging (MRI). A secondary objective is to compare strain and distensibility calculations using phase contrast (PC) and steady‐state free precession (SSFP) MRI sequences.

**Methods:**

Seven CMBPTs were tested under physiological aortic conditions with a pulsatile flow rate of 1650 mL/min using in vitro MRI. PC and SSFP sequences were employed, with PC estimating velocity and strain, while SSFP assessed strain separately. Image analysis was performed using QIR‐MR software.

**Results:**

Strain was measured using magnitude images. The mean maximum cross‐sectional areas were 419 ± 22.1 (PC) and 438.4 ± 24.3 mm^2^ (SSFP), while minimum areas were 374.5 ± 21.1 and 397.6 ± 23.4 mm^2^, respectively. The mean strain percentages were 12.1% ± 3.60% (PC) and 10.3% ± 3.20% (SSFP), with distensibility values of 1.5 ± 0.5 × 10^−3^ and 1.3 ± 0.4 × 10^−3^ mmHg^−1^, respectively.

**Conclusion:**

CMBPTs exhibit biomechanical properties similar to the abdominal aorta, suggesting potential for vascular applications. Both MRI sequences provided comparable results, supporting their reliability. Further research is needed to confirm long‐term performance.

## 1. Introduction

The use of autologous material for vessel replacement in infected fields is highly recommended in vascular surgery [1]. Current techniques employing cryopreserved aorta or neo‐aortoiliac systems are associated with various morbidities, including degeneration, early rupture, lower limb edema, risk of compartment syndrome, deep vein thrombosis, and extended operational duration [2–4]. These complications have prompted some surgeons to adapt their techniques by utilizing bovine pericardial patches (BPPs) to overcome these difficulties [5, 6]. The advantages of BPPs are consistent reliability, facility of utilization, strength, reduced suture line bleeding, and off‐the‐shelf availability [7]. Using the custom‐made bovine pericardial tube (CMBPT) as an alternative technique to the current recommended method of treatment of aortic graft infection, mycotic aortic aneurysm, or aorto‐enteric fistula has become an accepted method [1, 5, 6]. The BPP is characterized mechanically as a nonlinear, anisotropic, multilaminate composite material with pliability. It can be bent and twisted into various shapes, allowing it to undergo significant deformations during its physiological function as an implant or graft material [8]. Unfortunately, there is insufficient data to evaluate the functional biomechanical properties of the BPP, making it challenging to accurately estimate its performance in vivo. The primary objective of this study is to measure the circumferential physiological strain and distensibility of the CMBPT in vitro under simulated flow conditions of the abdominal aorta using magnetic resonance imaging (MRI) sequences. In addition, we aim to compare the physiological strain and distensibility using two types of 2D MRI sequences: phase contrast (PC) and steady‐state free precession (SSFP). Finally, we evaluated the velocity in the CMBPT using the PC sequence.

## 2. Materials and Methods

**FIGURE 1:**
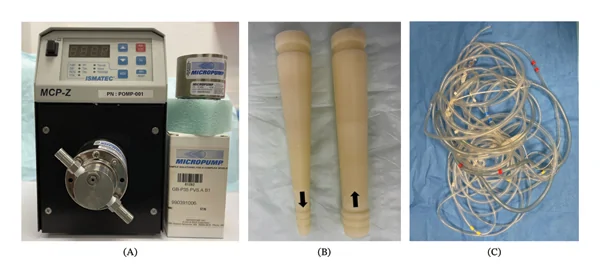
Used phantom material. (A) ISMATEC© MCP‐Z pump with pump head ISMATEC© MI0023 (Cole‐Parmer, Wertheim, Germany). (B) 3D‐printed sample, external dimension; 20 × 12 × 118 mm for inflow and 20 × 6 × 118 mm for outflow. (C) Silicone tube with different diameters to connect the pump with the CMBPT.

**FIGURE 2:**
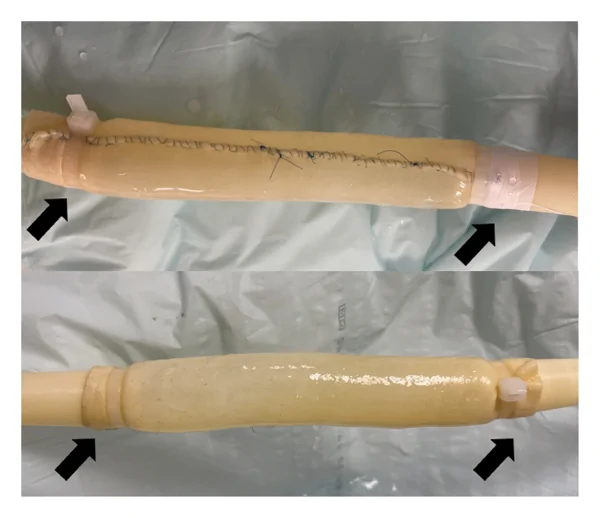
The CMBPTs were then attached to 3D‐printed connectors by nylon cable ties. Black arrow: nylon tie.

Experiments were conducted using a 3 T Magnetom Prisma MRI (Siemens Healthineers, Erlangen, Germany), utilizing an ISMATEC MCP‐Z pump (Cole‐Parmer, Wertheim, Germany) connected to an ISMATEC MI0023 pump head (Cole‐Parmer, Wertheim, Germany) (Figure 1A). The flow was progressively increased using silicone tubes with luminal diameters of 6, 10, and 15 mm, respectively; 3D‐printed connectors (with dimensions of 20 × 12 × 118 mm) were attached to the CMBPT inflow. For the return flow, we used a 3D‐printed connector to create outflow resistance simulating arterial resistance in human blood circulation and to maintain diastolic volume (Figures 1B and C). The outflow is poured into an open container and then absorbed by the same pump. Seven CMBPTs were constructed using a glutaraldehyde‐preserved Edwards BPP of 10 × 15 cm (Edwards Lifesciences, Irvine, CA, USA). The patch was sutured around a 20‐mm diameter syringe by Prolene 5‐0 using a running suture, and then a simple continuous suture was used along the running suture to have the final shape of the tube. The dimensions of CMBPT were 20 × 130 mm. The CMBPTs were then attached to 3D‐printed connectors by nylon cable ties (Figure 2).

**FIGURE 3:**
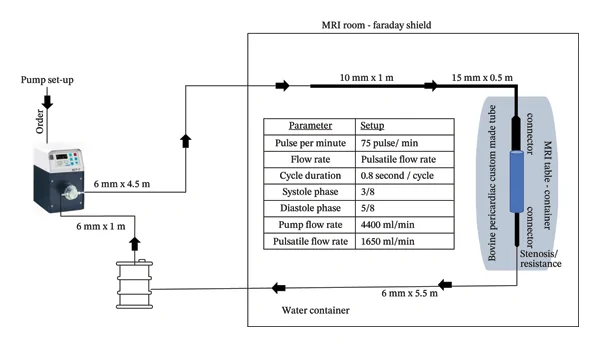
Illustration schema of the experience and the pump parameters.

The ISMATEC MCP‐Z pump (Cole‐Parmer, Wertheim, Germany) was placed outside the Faraday shield. We used a water‐open circulation with a pulsatile flow rate of 75 pulses/min. The pulsatile flow rate of the pump was 1650 mL/min, which is equivalent to the abdominal aorta flow rate (1602 ± 549 mL/min). The experience schema and pump setup are illustrated in Figure 3.

### 2.1. MRI Acquisition and Sequences

**FIGURE 4:**
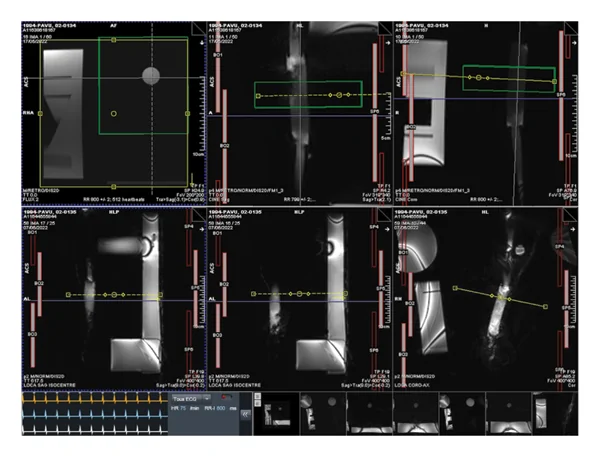
MRI processing interface of PC and SSFP sequences. The image shows the plane of interest in the axial, coronal, and sagittal axes of the CMBPT.

The acquisitions were performed in axial, longitudinal, and transversal planes of the CMBPT model. Two kinetic sequences were used. A PC sequence and an SSFP sequence were repeated three times for each CMBPT, resulting in a total of 21 samples. Four samples were excluded because of poor image quality (Figure 4). The acquisition settings were as follows: echo time of 1.7 msec, repetition time of 3.91 msec, acquisition matrix of 256 × 256, in‐plane pixel size of 1 × 1 mm^2^, and slice thickness of 5 mm. The PC acquisition with the destruction of residual transverse magnetization allowed obtaining a series of ultra‐fast and robust images with a very good signal‐to‐noise ratio. These acquisitions result in the reconstruction of two kinetic images: a series of magnitude images (PCm) and a series of corresponding phase‐contrast images (PCp). By applying bipolar gradient pulses perpendicular to the direction of flow, phase shifts are induced in moving protons, involving differences in the phase of the MRI signal and can be coded as the velocity of the blood flow. The magnitude images are created thanks to the magnitude of the flow‐compensated signal. The acquisition settings were as follows: echo time of 3.59 msec, repetition time of 6.17 msec, flip angle of 20°, acquisition matrix of 256 × 256, and in‐plane pixel size of 0.8 × 0.8 mm^2^ with a slice thickness of 5 mm. The encoding velocity was 100 cm/s. For both sequences, the acquisition was performed on the CMBPT with ECG synchronization (with an RR period of 800 msec) during the cardiac cycle (60 images per cycle).

#### 2.1.1. Experimentation Analysis

**FIGURE 5:**
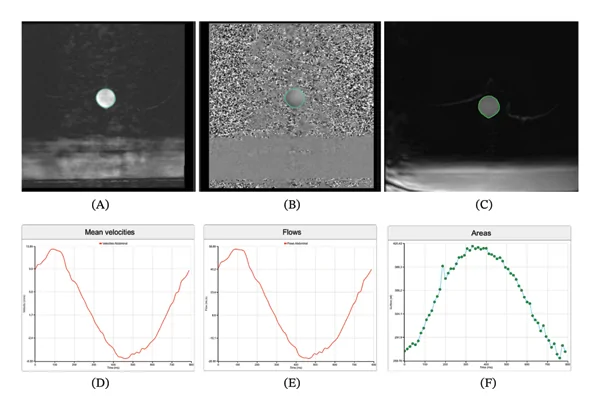
MRI analysis using QIR‐MR software (CASIS company, Quetigny, France). (A) PCm and (B) PCp images to evaluate the aortic strain percentage and the velocity. (C) SSFP sequence to evaluate the aortic strain percentage and the distensibility. (D) Mean velocity curve in cm/s obtained from PCm. (E) Flow curve in mL/s from PCp. (F) Surface area versus time curve in mm^2^ from SSFP.

For the analysis of the MRI images, the QIR‐MR software (CASIS company, Quetigny, France), which allows for detecting surface area, was used to calculate the velocity and distensibility. The contours of the CMBPTs were automatically detected, with the option for manual correction in both flow and anatomical sequences. The software was used to automatically identify the CMBPT contours and calculate their area during the cardiac cycle. The PC acquisitions were used to estimate both the velocity and the CMBPT strain. An additional SSFP sequence was used to increase the accuracy of CMBPT strain estimation (Figure 5). The pressure inside the CMBPT was measured using a central line connected to the pressure transducer machine before starting the experimentation. Pressure measurements were recorded at the distal end of the CMBPT and repeated five times. The average central flow pressure was calculated to be 80 ± 7.9 mmHg. The CMBPT strain and distensibility were measured by the aortic strain equation and distensibility equation as follows:

(1) aortic strain percentage=As−AdAd∗100,distensibility=As−AdPs−Pd∗Dd=ΔAΔP∗Ad,orΔACFP∗Ad,

where

 Ad: Luminal surface area at the diastolic phase.
 As: Luminal surface area at the systolic phase.
 CFP: Central flow pressure.
 Dd: Diameter of the vessel at diastole phase.
 Ds: Diameter of the vessel at the systolic phase.
 Pd: Pressure at the diastolic phase.
 Ps: Pressure at the systolic phase.

The velocity values have been measured in the axial plane in PCp. The peak systolic velocity and mean velocity values were measured for all samples.

The data obtained from the experimentation were analyzed using the Jamovi software (vVrsion 1.6.23.0, The Jamovi Project, Sydney, Australia).

## 3. Results

**FIGURE 6:**
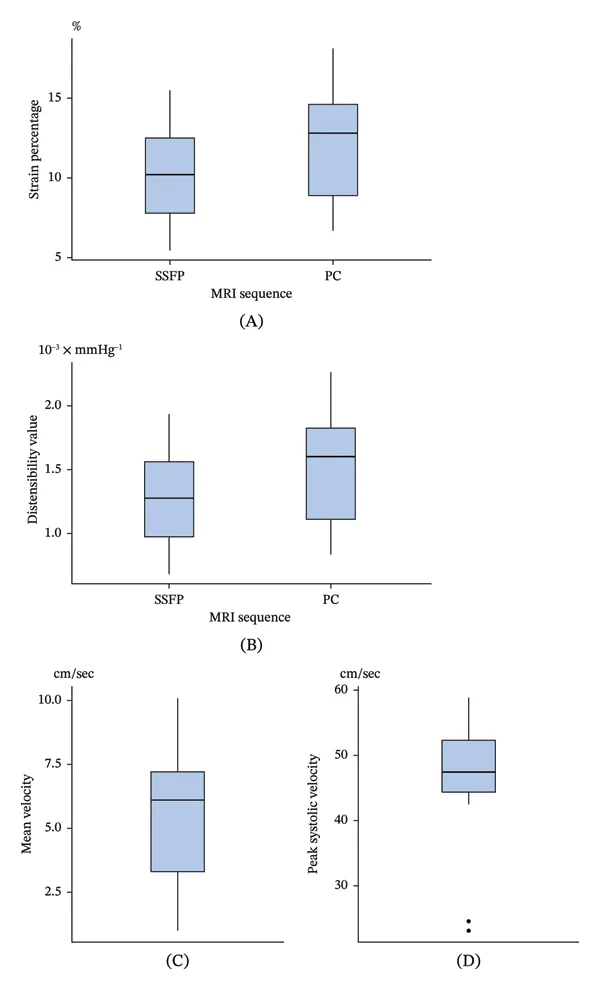
Box plot graphs. (A) Box plot for the strain percentage from the PCm and SSFP sequences. (B) Box plot of the distensibility value from the PCm and SSFP sequences. (C) Box plot of mean velocity values from the PCp. (D) Box plot of peak systolic velocity from the PCp.

The circumferential physiological strain was measured from two types of sequences: from the cross‐sectional areas obtained from PCm and from the cross‐sectional areas of SSFP. The mean of the maximum cross‐sectional area from the PCm is 419 ± 22.1 mm^2^ (Min: 376.9, Max: 450.4 mm^2^), while the mean of the minimum cross‐sectional area is 374.5 ± 21.1 mm^2^ (Min: 339.1, Max: 408.3 mm^2^). The mean of the maximum cross‐sectional area obtained from the SSFP is 438.4 ± 24.3 mm^2^ (Min: 385.9, Max: 465.7 mm^2^), while the mean of the minimum cross‐sectional area is 397.6 ± 23.4 mm^2^ (Min: 346.6, Max: 429.4 mm^2^). Given the small sample size, the Shapiro–Wilk p‐test was used to evaluate the distribution of our results. The Shapiro–Wilk test with a *p* value greater than 0.05 indicates a normal distribution of our results, allowing the use of the *t*‐test. The Shapiro–Wilk p‐test of strain percentage by PC sequence is 0.471, and for strain percentage by SSFP, the sequence is 0.503. The mean strain percentage calculated from the PCm sequence is 12.1% ± 3.60% (Min: 6.7%, Max: 18.1%), while the strain value obtained from SSFP is 10.3% ± 3.20% (Min: 5.5%, Max: 15.5%) (Table 1). The mean distensibility value obtained from the PCm sequence is 1.5 ± 0.5 × 10^−3^ mmHg^−1^ (Min: 0.8 × 10; Max: 2.3 × 10^−3^ mmHg^−1^). The distensibility value obtained from SSFP is 1.3 ± 0.4 × 10^−3^ mmHg^−1^ (Min: 0.7 × 10^−3^; Max: 1.9 × 10^−3^ mmHg^−1^). The Shapiro–Wilk p‐test for distensibility measured by PC and SSFP sequences is 0.471 and 0.503, respectively. The results are summarized in Table 1. The data obtained from the PC and SSFP sequences for strain evaluation were slightly different, along with the distensibility values (Figures 6A and B). Student′s *t*‐tests were used to compare the values obtained from the two sequences (strain percentage and distensibility). The Student′s *t*‐test found a nonsignificant difference between the SSFP and PC sequence data for strain and distensibilities (*p* value = 0.195).

**TABLE 1:** Strain percentage, distensibility, peak systolic velocity (PSV), and mean velocity obtained from PCm and SSFP sequences on 17 samples.

	Strain % PCm	Strain % SSFP	Distensibility PCm (10^−3^ × mmHg^−1^)	Distensibility SSFP (10^−3^ × mmHg^−1^)	PSV (cm/sec)	Mean velocity (cm/sec)
Mean	12.1 ± 3.60	10.3 ± 3.17	1.51 ± 0.450	1.29 ± 0.450	45.5 ± 11.0	5.73 ± 2.84
Minimum	6.69	5.47	0.836	0.684	23.1	1.00
Maximum	18.1	15.5	2.26	1.93	58.8	10.1
Shapiro–Wilk p	0.471	0.503	0.471	0.503		

The peak systolic velocity measured in our experiment is 45.5 ± 11 cm/sec, the maximum peak systolic velocity is 58.8 cm/sec, and the minimum peak systolic velocity is 23.1 cm/sec. The mean velocity value calculated in our study is 5.73 ± 2.84 cm/sec, while the maximum mean velocity is 10.1 cm/sec, and the minimum mean velocity is 1.0 cm/sec (Figures 6C and D).

## 4. Discussion

Our study aims to provide a better understanding of the biomechanical behavior of CMBPTs in simulated physiological conditions by providing physiological values of strain and distensibility.

The physiological strain value of the ascending and descending thoracic aorta ranges between 10% and 15%, whereas the abdominal aorta demonstrates a higher strain value ranging from 21% to 37% [9, 10]. Aortic strain decreases with age; individuals younger than 50 years exhibit strain values of 26% ± 9.46%, whereas those older than 50 years demonstrate lower values of 15.1% ± 7.29%^12^. The prosthetic grafts exhibit markedly lower strain values, approximately 1.8% for Dacron grafts and 1.2% for expanded polytetrafluoroethylene grafts [11].

In this study, the CMBPT strain value obtained is 12.1% ± 3.60% for the PCm approach and 10.3% ± 3.20% for the SSFP approach. Our results are within the normal range of the normal aortic strain measured by the MRI technique [12]. They also estimated the distensibility value by age groups: 7.44 ± 2.54 × 10^−3^ mmHg^−1^ for people less than 50 years old and 2.89 ± 1.29 × 10^−3^ mmHg^−1^ for people more than 50 years old [12]. Voges et al. found a value of 8.3 ± 3.0 × 10^−3^ mmHg^−1^ [13]. Rose et al. confirmed that distensibility decreases with age and found that women have higher aortic distensibility than men at the level of the descending aorta (7.20 ± 1.61 × 10^−3^ vs. 5.05 ± 2.40 × 10^−3^ mmHg^−1^) [14].

The reported strain values may vary depending on the measurement methodology, including the specific MRI sequences employed for strain assessment. Herment et al. found in their study significant differences in aortic strain values between the two methods, SSFP and PC sequences, used to estimate the strain values (*p* < 0.02). The values were 17.8% ± 11.7% for the SSFP sequence and 16.9% ± 8.18% for the PCm sequence [15]. This difference is not confirmed in our study, which could be explained by the small sample size.

According to our results, the estimation of the CMBPT physiological strain can be calculated from both SSFP and PC acquisitions. Small variations across acquisition methods were approved in the study of Hermet et al., mostly as a result of poor image quality or artifacts that can impede the segmentation process [15]. Therefore, when a local evaluation of aortic stiffness is required, better results were obtained from SSFP sequences compared to PC sequences. The balanced SSFP sequence provides the highest signal‐to‐noise ratio compared to other sequences, but it has limited practical use for diagnostic imaging because of the mixed T2/T1 contrast behavior. However, SSFP is widely used in functional and morphological imaging in the cardiovascular field. Using the PC sequence, on the other hand, has a complementary role to the SSFP sequence, where it could provide additional flow information and the ability to precisely evaluate velocity (in particular peak wave velocity) to determine aortic stiffness segment [15, 16].

In our study, the central CMBPT pressure was calculated by a central line inserted into the distal third of the CMBPT (80 mmHg), while the cross‐sectional areas were obtained from both PC and SSFP. We found that the mean distensibility value obtained from the PCm sequence is 1.5 ± 0.5 × 10^−3^ mmHg^−1^, while the distensibility value obtained from SSFP is 1.3 ± 0.4 × 10^−3^ mmHg^−1^. There are no significant differences between SSFP and PCm distensibility values in our study.

The mechanical behavior of bovine pericardium is predominantly determined by its collagen‐dense hierarchical microstructure, marked intrinsic anisotropy, and variability related to both the anatomical site of harvesting and the age of the animal [17–19].

Our findings indicate that CMBPTs may exhibit mechanical properties that are intermediate between native aortic tissue and conventional prosthetic grafts, thereby potentially mitigating the degree of compliance mismatch.

The small sample size has an impact on the measured data. These findings require further investigation in a larger cohort to have more accurate results. Performing similar experiments on different BPP brands approved in the medical field would give more accurate results for biomechanical parameters. The pump‐generated flow was designed to reproduce abdominal aortic flow rates; the model does not capture the full spectrum of physiological pressure waveforms, vascular branching patterns, or dynamic interactions with surrounding tissues.

To our knowledge, no previous publication has evaluated the biomechanical properties of CMBPTs using MRI in vitro, which represents the main strength of our study.

## 5. Conclusion

This study presents an in vitro MRI‐based assessment of the biomechanical behavior of CMBPTs under simulated abdominal aortic flow conditions. The circumferential strain values obtained were within the range reported for the native thoracic aorta, while distensibility values were lower than those reported for the native abdominal aorta in vivo. These findings suggest that CMBPTs may exhibit intermediate biomechanical properties between native aortic tissue and conventional prosthetic grafts, although direct comparison with in vivo conditions is limited by the constraints of the in vitro model. No significant difference was found between SSFP and PC acquisitions for strain and distensibility estimation, supporting the use of either sequence in future phantom studies. These results are preliminary and should be interpreted with caution given the small sample size, the use of a simplified pressure model, and the absence of direct systolic and diastolic pressure measurements. Further biomechanical and clinical studies are required to validate the performance of CMBPTs under true physiological conditions.

## Funding

This research received no external funding.

## Conflicts of Interest

Thomas Decourselle is affiliated with the CASIS company. The remaining authors declare no conflicts of interest.

## Data Availability

The data that support the findings of this study are available from the corresponding author upon reasonable request.
